# scMNMF: a novel method for single-cell multi-omics clustering based on matrix factorization

**DOI:** 10.1093/bib/bbae228

**Published:** 2024-05-16

**Authors:** Yushan Qiu, Dong Guo, Pu Zhao, Quan Zou

**Affiliations:** School of Mathematical Sciences, Shenzhen University, 518000, Guangdong, China; School of Mathematical Sciences, Shenzhen University, 518000, Guangdong, China; College of Life and Health Sciences, Northeastern University, Shenyang, 110169, China; Institute of Fundamental and Frontier Sciences, University of Electronic Science and Technology of China, Chengdu, 610056, China

**Keywords:** single-cell multi-omics, non-negative matrix factorization, joint learning

## Abstract

**Motivation:**

The technology for analyzing single-cell multi-omics data has advanced rapidly and has provided comprehensive and accurate cellular information by exploring cell heterogeneity in genomics, transcriptomics, epigenomics, metabolomics and proteomics data. However, because of the high-dimensional and sparse characteristics of single-cell multi-omics data, as well as the limitations of various analysis algorithms, the clustering performance is generally poor. Matrix factorization is an unsupervised, dimensionality reduction-based method that can cluster individuals and discover related omics variables from different blocks. Here, we present a novel algorithm that performs joint dimensionality reduction learning and cell clustering analysis on single-cell multi-omics data using non-negative matrix factorization that we named scMNMF. We formulate the objective function of joint learning as a constrained optimization problem and derive the corresponding iterative formulas through alternating iterative algorithms. The major advantage of the scMNMF algorithm remains its capability to explore hidden related features among omics data. Additionally, the feature selection for dimensionality reduction and cell clustering mutually influence each other iteratively, leading to a more effective discovery of cell types. We validated the performance of the scMNMF algorithm using two simulated and five real datasets. The results show that scMNMF outperformed seven other state-of-the-art algorithms in various measurements.

**Availability and implementation:**

scMNMF code can be found at https://github.com/yushanqiu/scMNMF.

## INTRODUCTION

Cells, which are the basic components of all living things, perform vital biological functions that keep life running normally. Numerous unique cell types with varying roles make up multicellular organisms. The structure and function of tissues can be understood by classifying the different cell types [1]. However, because an exact and uniform description of cell types is still lacking, identifying cell types remains difficult. Further advancements in bioinformatics have enabled the analysis of transcriptome data of individual cells. Single-cell transcriptomic analysis technology dates back to 2009 [2] and has been used extensively to analyze single-cell RNA sequencing (scRNA-seq) data. The accumulation of scRNA-seq data offers the chance to investigate the characteristics and actions of diverse cell types, and scRNA-seq methods for cell classification have been developed based on transcriptomic similarity. However, the numerous drawbacks of scRNA-seq make cell clustering very challenging [3]. In particular, sequencing technologies for single-cell transcriptome data pose certain dimensionality issues, because they disrupt the distinct expression profiles within an entire genome. Therefore, relying solely on intercellular similarity is one-sided, because the distance differences would have already changed. The analysis tools for single-cell multi-omics data have become increasingly mature and have provided significant impetus for biological research. Single-cell studies differ from traditional bulk methods in that they can handle data heterogeneity. Clustering, as an unsupervised learning method [4], can group similar single cells into the same cluster, thereby detecting differences and similarities among cells [5], and inferring biological information. In most single-cell studies, clustering analysis is a crucial and well-researched phase. Biological processes can be investigated by clustering data, which cannot be achieved by studying large datasets alone [6]. However, clustering analysis for single-cell multi-omics data remains a challenging problem. With the rapid development of various clustering analysis techniques, existing algorithms can be broadly classified as early fusion, late fusion and intermediate fusion algorithms.

Early fusion is the simplest of the three methods, where all omics matrices are concatenated, and single-omics clustering algorithms are applied to the merged matrix [7, 8]. Representative methods of early fusion include ICluster [9], which concatenates multi-omics data and then uses probabilistic modeling to reduce the dimensionality of the data, and LRACluster [10], which builds a low-rank approximation model based on multi-omics data to identify their common subspace and subsequently perform clustering operations. However, early fusion methods have several drawbacks: (i) if not correctly standardized, they may give greater weight to omics that have greater characteristics, (ii) they ignore the various data distributions across various omics and (iii) in situations where the data dimensionality is already high, they further increase the data dimensionality, making it more difficult to differentiate between samples.

Late fusion involves partitioning using different clustering methods on different omics datasets. The advantage of this method is that any clustering algorithm can be applied to each omics dataset [11, 12]. However, the disadvantage is that if the clustering results are obtained by integration, the weaker information in each omics dataset may be lost. Late fusion methods include SAME-clustering [11], which is based on graph partitioning for clustering integration, and KLIC [13], which integrates clustering through kernel learning.

Intermediate fusion is distinct from the other two fusion methods and primarily has three categories. Methods in the first category use similarity-based methods, where clustering is performed by constructing a similarity matrix between data points. CiteFuse [14] is one such method that calculates similarity matrices among different omics data, then merges the matrices using a fusion method and finally clusters the merged similarity matrix. Seurat [15] is another method in this category. Methods in the second category are based on dimensionality reduction model algorithms, which assume that there is an invariant low-dimensional structure between the data, and the low-dimensional structures correspond to the number of clusters. Methods in this category include CoupledNMF [16], MOFA+ [17] and TotalVI [18]. The third category of methods is statistical algorithms based on data modeling. The advantage of these models is that they can utilize biological information while determining the distribution functions. Representative methods in this category include BREM-SC [19] and Clonealign [20]. Similarity-based methods cannot explicitly consider dropout events and, for different omics data, they simply concatenate the data features or remember them directly, and therefore cannot fully exploit the information between different omics. Most statistical-based methods require data to have a specific distribution, but this may not necessarily be applicable in practical applications.

Inspired by the problems associated with the methods described above, we developed a non-negative matrix factorization (NMF)-based method that differs from the traditional approach of separate dimensionality reduction and clustering processes. The aim was to better analyze the intrinsic correlations between different omics data and investigate the mutual influences between joint dimensionality reduction and clustering. To extract the intrinsic correlations between different omics data, we used NMF to process the data and obtain their common feature matrix. Then, we employed joint dimensionality reduction to study the clustering results using different omics datasets. By integrating dimensionality reduction and clustering analysis, our algorithm demonstrates the advantage of iterative influence between feature selection in dimensionality reduction and cell clustering, leading to a significant improvement in clustering performance.

## MATERIALS AND METHODS

### Model description

We take $\mathit{M}$ matrices $\mathbf{X}_{1}, \ldots \mathbf{X}_{M}$ as inputs, representing $\mathit{M}$ omics datasets. $\mathbf{X}_{k}\in R^{ n \times J_{k}}$, where $n$ represents the count of cells and $J_{k}$ represents the count of features for block $k$. In our method, we offer a model built using methods for matrix factorization, i.e. 


(1) \begin{align*}& {X}_{k} \approx W {H}_{k},\end{align*}

where $\mathit{W}\in R^{ n\times p }$ is a shared basis matrix that allows simultaneous clustering of cells across $\mathit{M}$ omics blocks. $\mathit{H_{k}} \in R^{ p\times J_{k}}$ is the coefficient matrix for block $k$. The quantity of latent variables in this model is denoted by the variable $p$. Here, we impose non-negativity and sparsity constraints on matrix $\mathit{W}$ to ensure interpretability of the model. $H_{k}$ is subject to a sparsity constraint to carry out variable selection concurrently with cell clustering. A deeper understanding of the factors influencing the cell clustering is ensured by sparsity. Then, the following is an extension of Equation (1): 


(2) \begin{align*}& \begin{split} R_{W, H_{1}, \ldots, H_{M}}&=\sum_{k=1}^{M}\left\|X_{k}-WH_{k}\right\|^{2}+\lambda_{k}\left\|H_{k}\right\|_{1}+\sum_{i=1}^{n} \mu_{i}\left\|w_{i\cdot}\right\|_{1} \\ & \qquad \qquad \qquad \qquad \text{ s.t. } W \geqslant 0, \end{split}\end{align*}

where $w_{i\cdot }$ is the $i$th row, $\|\cdot \|$ is the Frobenius parametrization and $\|\cdot \|_{1}$ is the $l_{1}$-norm. Both $\lambda _{k}$ and $\mu _{i}$ are sparse constraint parameters. $B$ and $F$ are both non-negative, and their product is used to further estimate the decomposition matrix $W$ to gain the possible characteristics of every single cell. 


(3) \begin{align*}& W \approx B F, \quad \text{ s.t.} B \geqslant 0, F \geqslant 0,\end{align*}

where $\mathit{B}\in R^{ n \times k_{1}} $ and $\mathit{F}\in R^{ k_{1} \times p }$ are the feature and basis matrix, and $k_{1}$ is the number of cell types. Equation (3) can be solved by minimizing the approximation, i.e. 


(4) \begin{align*}& Q_{B,F} = \left\|W-BF\right\|^{2}, \text{ s.t.} B \geqslant 0, F \geqslant 0.\end{align*}

Nevertheless, NMF is unable to unveil the intrinsic geometric arrangement of cells, such as manifold embeddings within high-dimensional spaces. We expect $\mathit{B}$ to preserve the inherent geometric structure of matrix $\mathit{W}$ [21]. In particular, if two cells, denoted as ‘$c_{i}$’ and ‘$c_{j}$’, are close together in the initial space, their representations in the matching space, i.e. $s_{i}$ and $s_{j}$, should also exhibit proximity, and vice versa. We construct a Laplacian graph $\mathit{G}$ to represent the close relationships between cells contained in matrix $\mathit{W}$. The vertices of graph $\mathit{G}$ describe cells, while the edges represent the similarity between two vertices. Let $\mathit{A_{ij}}$ denote the weight matrix, and given that $\mathit{U_{p}}\left (c_{i}\right )$ represents the $p$ nearest neighbors of $c_{i}$, we have 


(5) \begin{align*}& A_{i j}= \begin{cases}\mathrm{e}^{-\frac{\left\|c_{i}-c_{j}\right\|^{2}}{\sigma},}, & c_{i} \in U_{p}\left(c_{j}\right) \text{ or}\ c_{j} \in U_{p}\left(c_{i}\right) \\ 0, & \text{ others }.\end{cases}\end{align*}

We denote $L=D-A$ as the Laplacian matrix of graph $\mathit{G}$, where $D_{i i}=\sum _{j} A_{i j}$ is the diagonal matrix. Cai *et al.* [22] demonstrated that trace optimization can be used to define the local topological structure preservation, i.e. 


(6) \begin{align*}& \frac{1}{2}\sum_{i,j}a_{ij}||s_{i}-s_{j}||^{2}=\operatorname{Tr}\left(BLB^{T}\right).\end{align*}

Thus, binding the representation of Equation (6), the objective function of clustering can be defined as 


(7) \begin{align*}& Q_{B,F} = \left\|W-BF\right\|^{2}+\alpha \operatorname{Tr}\left(BLB^{T}\right), \text{s.t.} B \geqslant 0, F \geqslant 0.\end{align*}

and $\alpha $ decides the significance of the regularization. Finally, by combining Equations (2) and (7), we obtain the final objective function of the joint analysis model as 


(8) \begin{align*}& \begin{aligned} \min O= & R_{W, H_{1}, \cdots H_{M}}+Q_{B, F} \\ = & \sum_{k=1}^{M}\left\|X_{k}-W H_{k}\right\|^{2}+\lambda_{k}\left\|H_{k}\right\|_{1}+\sum_{i=1}^{n} \mu_{i}\left\|w_{i}\right\|_{1} \\ & +\|W-B F\|^{2}+\alpha \operatorname{Tr}\left(B L B^{\top}\right)\\ & \text{s.t.} W \geqslant 0, B \geqslant 0, F \geqslant 0. \end{aligned}\end{align*}

**Figure 1:**
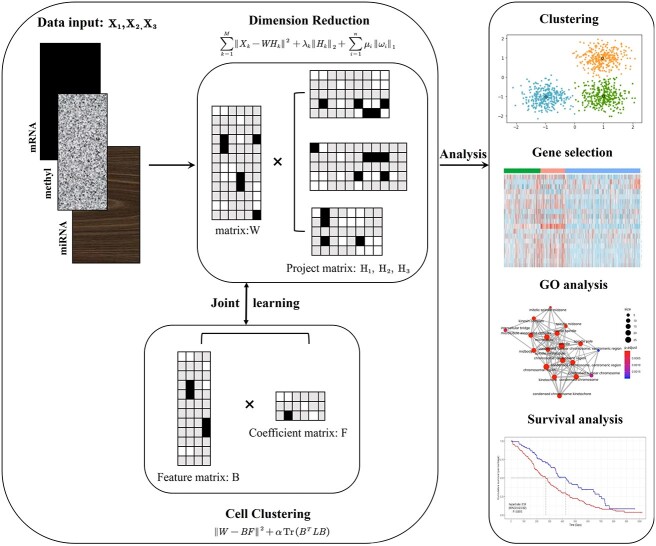
Overview of the scMNMF model. This is a joint analysis model that differs from the traditional approach of separately conducting dimensionality reduction and clustering.

The framework of the method we constructed is illustrated in Figure 1.

### Optimization of the algorithm

Taking into account the non-convexity of the objective function, we employ an alternating iterative algorithm to optimize it, by fixing all other variables and making the optimization problem of the remaining variable convex. The algorithm continues to iterate until it converges or reaches a stopping condition. The update details of Equation (8) are provided in Supplementary Section 1 (see Supplementary Data available online at http://bib.oxfordjournals.org/). The convergence analysis of the updating rule is provided in Supplementary Section 2 (see Supplementary Data available online at http://bib.oxfordjournals.org/).

### Related parameters

scMNMF has a total of five parameters. Parameter $p$ is the count of features after dimensionality reduction, $k_{1}$ is the count of cell clusters and $\lambda _{k}$, $\mu _{i}$, and $\alpha $ determine the importance of the corresponding sparsity constraint and regularization. The method for selecting these five parameters is detailed in Supplementary Section 3 (see Supplementary Data available online at http://bib.oxfordjournals.org/).

## PERFORMANCE EVALUATION

### Accuracy

ACC is a commonly used performance metric in machine learning and data analysis, which measures the percentage of correctly classified instances in a dataset. 


(9) \begin{align*}& ACC=\max_{m} \frac{\sum_{i=1}^{k} 1\left\{l_{i}=m\left(u_{i}\right)\right\}}{k},\end{align*}

where $k$ represents the count of cells, $l_{i}$ represents the true labels and $m(\cdot )$ denotes all possible one-to-one mappings between cluster assignments and true labels.

### Adjusted rand index

Adjusted rand index (ARI) is a metric used to measure the consistency between clustering results and true labels, and its definition involves the matching of pairs of samples in the clustering results. 


(10) \begin{align*}& ARI=\frac{\left(\begin{array}{l} k \\ 2 \end{array}\right)(s+n)-[(s+t)(s+m)+(n+t)(n+m)]}{\left(\begin{array}{l} k \\ 2 \end{array}\right)-[(s+t)(s+m)+(n+t)(n+m)]},\end{align*}

where $k$ is the total number of cells, $s$ is the number of cell pairs with the same cell label corresponding to the same cluster, $t$ is the number of cell pairs with different cell labels corresponding to different clusters, $m$ is the number of cell pairs with the cell labels corresponding to different clusters and $n$ is the number of cell pairs with different cell labels corresponding to the same cluster.

### Normalized mutual information

Mutual Information ($MI$) is defined as follows: 


(11) \begin{align*}& MI=\sum_{s} \sum_{t} f(s, t) \log \frac{f(s, t)}{f(s) f(t)},\end{align*}

where $f(s,t)$ is the joint distribution of random variables $S$ and $T$, and $f(s)$ and $f(t)$ are the marginal distributions of $S$ and $T$. NMI is defined as 


(12) \begin{align*}& NMI = \frac{2MI}{h(P)+h(Q)},\end{align*}

where $h(P)$ and $h(Q)$ represent the entropy of the clustering result, denoted as $P$, and the true labels, denoted as $Q$, respectively.

### Adjusted mutual information

Adjusted mutual information (AMI) measures the similarity between cluster assignments and true labels, defined as 


(13) \begin{align*}& AMI=\frac{MI-E[MI]}{\max (h(P), h(Q))-E[MI]},\end{align*}

where $MI$ is defined as above, where $E[MI]$ is its mathematical expectation, and $h(P)$ and $h(Q)$ are the entropies of the predicted labels and true labels, respectively.

## EXPERIMENTAL RESULTS

### Datasets

We used two simulated and five real datasets to evaluate the performance of the scMNMF method. We preprocessed all datasets, and specific steps can be found in Supplementary Section 4 (see Supplementary Data available online at http://bib.oxfordjournals.org/). Detailed information about these datasets is provided in Table 1.

- Simulated datasets We generated two single-cell multi-omics datasets (Sim1 and Sim2), each comprising gene expression omics and epigenetics omics data [23]. Sim1 contained 530 cells with 3 cell types, and Sim2 contained 250 cells with 5 cell types.
- 10X_10K peripheral blood mononuclear cell (PBMC) dataset The PBMC dataset was obtained from the 10x Genomics website. Each cell in this dataset has matched scRNA-seq and antibody-derived tags (ADT) data. The cell types for each sample were determined from biological information of protein and gene markers. The dataset includes 6661 cells representing 7 different cell clusters.
- PBMC Specter dataset The Specter dataset consists of 3762 cells, divided into 16 cell types. The true labels in this dataset are from Specter [24]. The dataset can be obtained from GitHub (https://github.com/canzarlab/Specter).
- Spleen & Lymph nodes (SLN111) dataset Specific information about the spleen dataset and cell type labels can be found in the TotalVI method [18].
- SMAGE dataset The SMAGE dataset consists of 11 020 cells, divided into 12 cell types. It includes two types of omics data. The scRNA-seq data can be used directly for subsequent analysis, whereas the ATAC gene counts were obtained using tools from Lin *et al.* [25].
- Human bone marrow mononuclear cell (BMNC) dataset The BMNC dataset contains of cells from eight donors and was curated by the Human Cell Atlas. The cell type labels for this dataset can be obtained using Seurat software [15].

**Table 1:** Summary of the two simulated datasets and five real datasets

**Dataset**	**Cell**	**RNA**	**ADT**	**ATAC**	**Type**
Sim1	$530$	$2000$		$5000$	$3$
Sim2	$250 $	$2500$		$5000$	$5$
Specter	$3762$	$33\,538$	$49$		$16$
10X_10K	$6661$	$33\,538$	$17$		$7$
SMAGE	$11\,020$	$36\,611$		$20\,010$	$12$
Spleen	$16\,828$	$13\,553$	$112$		$35$
BMNC	$30\,672$	$17\,009$	$25$		12

Type, number of cell types.

### Methods used for performance comparisons

We compared our scMNMF method with seven advanced methods.

- SCMDC SCMDC [25] is a deep learning model that processes different omics datasets in an end-to-end manner and then conducts clustering analysis for the next step based on jointly learned latent deep embedding features.
- MoClust MoClust [26] is a newly proposed efficient clustering method for handling data. It introduces modality-specific autoencoders to describe multi-omics data and employs distribution alignment with contrastive learning to flexibly merge the omics blocks into omics-invariant representations.
- Seurat After reducing the data to a lower-dimensional space, Seurat [15] constructs a weighted nearest neighbor graph and then uses the Leiden algorithm or other related algorithms to iteratively optimize and merge cells together.
- Tscan Tscan [27] is a traditional clustering method that processes omics data by reducing dimensionality through principal component analysis and then applies a mixture model to the reduced data for further clustering analysis.
- BREM-SC BREM-SC [19] is one of the earliest models proposed for clustering analysis of CITE-seq data, capable of characterizing different omics data. This method conducts clustering analysis by establishing Bayesian models for two blocks of omics data.
- CiteFuse CiteFuse [14] independently calculates similarity matrices between two different omics datasets and then applies an efficient fusion algorithm to merge the matrices based on this similarity. Finally, the merged similarity matrix undergoes clustering analysis based on existing graph fusion algorithms.
- TotalVI TotalVI [18] uses a variational autoencoder to process two different single-cell multi-omics datasets, then evenly distributes the learned modal encoder data and finally uses algorithms for subsequent clustering analysis.

### Clustering performance

**Figure 2:**
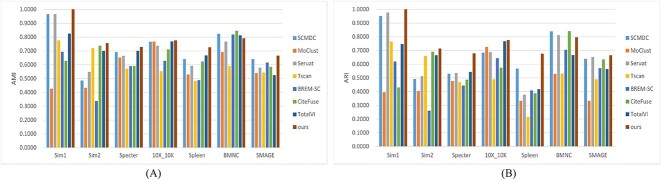
Clustering performance of different methods on two simulated datasets and five real datasets. (**A**) Performance measured by AMI. (**B**) Performance measured by ARI.

We used four popular performance metrics, ACC, ARI, AMI and NMI, to compare the clustering results. The clustering performance of seven algorithms and scMNMF on the two simulated datasets and five real datasets measured by AMI and ARI is shown in Figure 2. The clustering performance measured by ACC and NMI is given in Supplementary Figure 1 (see Supplementary Data available online at http://bib.oxfordjournals.org/).

Figure 2 shows that scMNMF outperformed the other seven competing methods on all the datasets, except the BMNC dataset. The alternating influence of feature selection in dimensionality reduction and cell clustering of the scMNMF algorithm significantly improved its clustering performance. Tscan [27] produced the worst performance across the five real datasets, indicating that its traditional single-cell clustering method was unable to fully leverage the advantages of multi-omic data to enhance clustering performance and was not suitable for multi-omics data. However, Tscan performed quite well on the simulated datasets, further indicating that traditional clustering methods, including the one implemented in Seurat [15], are still somewhat unstable when dealing with single-cell multi-omics data. Although BREM-SC [19] is a mature clustering analysis algorithm, the results showed that it performed poorly on the seven datasets we ran. Its poor performance can be attributed to several limitations of the BREM-SC algorithm, particularly its inability to represent dropout events. SCMDC [25] and TotalVI [18] produced better clustering metrics than the other methods on all seven datasets, indicating that deep learning algorithms have an advantage in handling high-dimensional data. Our scMNMF model did not perform well on the BMNC dataset, whereas CiteFuse [14] produced the best performance on this dataset. This difference in performance may be because CiteFuse [14] is sensitive to high computational complexity, whereas scMNMF requires iterative optimization for the optimal solution, resulting in suboptimal performance when dealing with large-scale datasets such as BMNC. For ACC, scMNMF performed better than the other seven algorithms on the two simulated datasets and all the real datasets, except the BMNC dataset (Supplementary Figure 1, see Supplementary Data available online at http://bib.oxfordjournals.org/). For NMI, the scMNMR model ranked in the top two for all the datasets, except the Specter dataset. Overall, scMNMF performed well on all the datasets compared the performances of the other methods tested.

**Figure 3:**
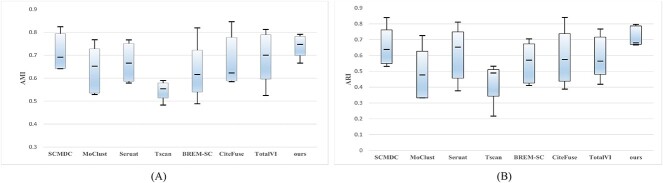
Boxplot of scMNMF and other related algorithms on five real datasets. (**A**) Performance measured by AMI. (**B**) Performance measured by ARI.

**Figure 4:**
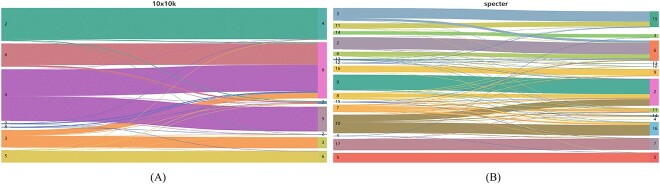
Sankey plots for two of the real datasets. (**A**) The 10X_10K dataset. (**B**) The Specter dataset. The numbers on the left of the plots indicate the true label categories, and the numbers on the right of the plots indicate the predicted label categories.

To further validate the feasibility of our model on five real datasets, we generated box plots based on the clustering results, to showcase the overall clustering effect of scMNMF and the other comparative methods. We calculated the lower quartile, maximum value, minimum value, median and upper quartile of the clustering metric scores for each method across different datasets. Then, we visualized the obtained values in a box plot to compare the overall performance of the methods. The height of the box indicates the interquartile range (IQR). A small IQR indicates concentrated and stable clustering results, whereas a large IQR indicates unstable clustering results, implying the method is unstable. The results for AMI and ARI are shown in Figure 3, and the results for ACC and NMI are in Supplementary Figure 2 (see Supplementary Data available online at http://bib.oxfordjournals.org/). The plots show that scMNMF produced relatively concentrated performances across all datasets, indicating that its overall performance was better than those of the other methods tested. We created Sankey diagrams to illustrate the cluster assignments (Figure 4, the diagrams for the other datasets are given in Supplementary Figure 3, see Supplementary Data available online at http://bib.oxfordjournals.org/). When the number of labels was small, the clustering results were more pronounced. However, when the dataset contains a larger number of cell types, including some sparsely represented cells, there may be a certain degree of misalignment, resulting in suboptimal classification results. Nevertheless, overall, our scMNMF model produced a relatively advantageous clustering performance across different datasets.

**Figure 5:**
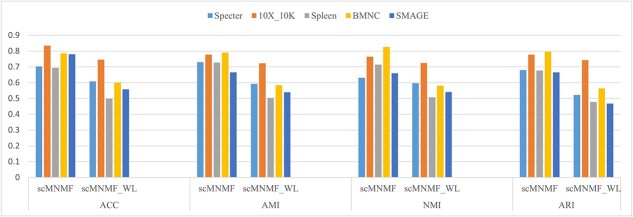
Clustering performance of scMNMF with and without Laplace regularization on five real datasets. scMNMF, with Laplace regularization; scMNMF_WL, without Laplace regularization.

We further investigated whether Laplacian regularization improved the clustering performance. Because Laplacian regularization affected the iterative formulation of the feature matrix $B$, we modified the iterative formula and conducted clustering performance comparison experiments on the five real datasets. The results indicate that when the Laplacian regularization term was added, the clustering performance of the scMNMF algorithm significantly improved compared with its performance without the Laplacian regularization term (Figure 5). This result suggests that Laplacian regularization effectively preserved the intrinsic geometric structure of the feature matrix, thereby enhancing clustering performance.

**Figure 6:**
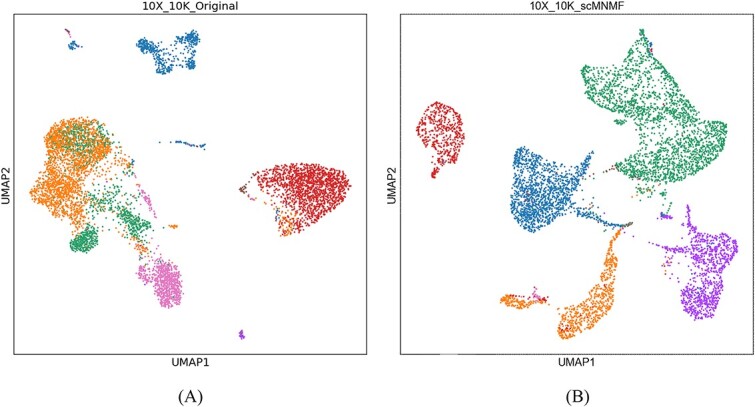
UMAP visualization of the clustering performance of scMNMF with the 10X_10K dataset. (**A**) Original 10X_10K dataset. (**B**) 10X_10K dataset after clustering by scMNMF.

We used UMAP [28] to visualize the clustering performance. Taking the 10X_10K dataset as an example (Figure 6), the results show that cells were intertwined in the original data, and after processing with scMNMF, the cells were effectively separated. Similar effects were observed for the other datasets (Supplementary Figure 4, see Supplementary Data available online at http://bib.oxfordjournals.org/). Thus, we demonstrated that our scMNMF model can indeed help to improve the cell clustering.

### Downstream analysis

We performed some downstream analysis of the scMNMF results, including gene ontology (GO) [29] and survival analysis. Gene expression is closely related to the characteristics and morphology of cells, so identifying the relevant informative genes is important. The specific steps that we used to select informative genes are provided in Supplementary Section 5 (see Supplementary Data available online at http://bib.oxfordjournals.org/). We identified 28 informative genes from the 10X_10K dataset that were differentially expressed. Details of the 28 genes are listed in Supplementary Table 1 (see Supplementary Data available online at http://bib.oxfordjournals.org/).

**Figure 7:**
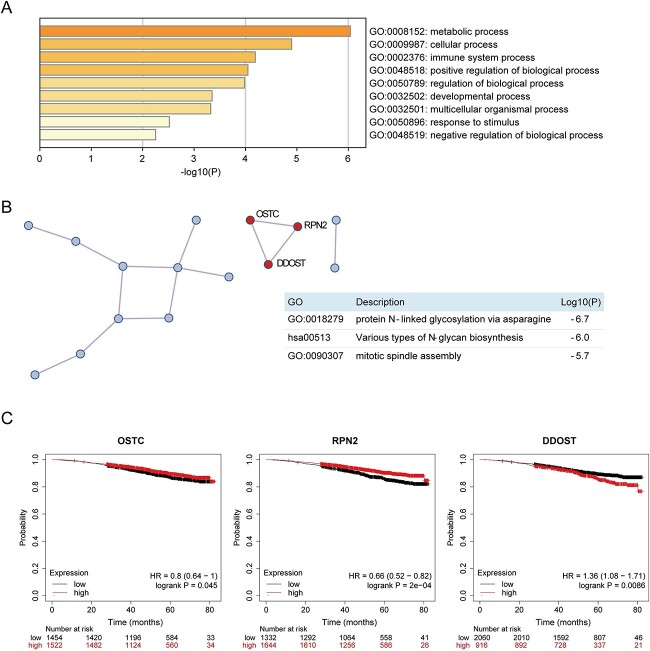
Downstream analysis of the scMNMF results. (**A**) Functional enrichment analysis of marker genes based on GO. (**B**) Protein–protein interactions of the marker genes. Molecular Complex Detection was used to identify a molecular complex (i.e., OSTC` RPN2 and DDOST). (**C**) Kaplan–Meier survival analysis for marker genes *OSTC*, *R*PN2 and *D*DOST.

To evaluate the biological significance of the 28 genes, we performed GO and KEGG pathway enrichment analysis as described previously [30]. Briefly, we considered all the genes in the genome as enrichment references. GO terms with $P$-values ¡0.01, minimum count of 3 and enrichment factor ¿1.5 were grouped based on the similarity of their members. The results of GO analysis are shown in Figure 7A. Carbohydrate derivative biosynthetic process, protein N-linked glycosylation via asparagine, mitotic spindle assembly, lymphocyte mediated immunity, *in utero* embryonic development, cell activation, wound healing, regulation of post-translational protein modification and angiogenesis were significantly enriched. Among the enriched GO terms, lymphocyte mediated immunity, wound healing and regulation of angiogenesis play pivotal roles in tumor invasion and metastasis. Protein–protein interaction (PPI) enrichment analyses were carried out using STRING [31], BioGrid [32], OmniPath [33] and InWeb IM, and the results are shown in Figure 7B. The protein complex screened by the PPI analysis plays an important role in protein N-linked glycosylation, and N-linked glycosylation of PD-L1 at N35, N192, N200 and N219 is required for PD-L1-dependent tumor metastasis, implying the involvement of the three genes in the complex (informative genes) in tumor progression and tumor immune escape. To analyze the correlation between the informative genes and the survival time of patients, we used the Kaplan–Meier tool (https://kmplot.com) to plot survival curves for the three informative genes, then analyzed their impact on survival. We found that out of the 28 selected informative genes, 22 were correlated with patient survival time (Figure 7C and Supplementary Figure 5, see Supplementary Data available online at http://bib.oxfordjournals.org/). The molecular complex composed of OSTC, RPN2 and DDOST is involved in protein N-linked glycosylation and regulates the survival of patients with breast cancer. OSTC and RPN2 promote patient survival, whereas DDOST shortens survival (Figure 7C). In summary, scMNMF can effectively facilitate research on breast cancer progression and is a highly effective tool for biological predictions.

## CONCLUSION

Cell clustering has become an important and rapidly developing direction in single-cell research in recent years. Clustering combines different types of single-cell data, such as gene expression, protein expression and chromatin states, to comprehensively classify and cluster individual cells. This integrated analysis provides a comprehensive cell-type identification and functional interpretation, which aids in the understanding of the complex biological characteristics of cells. Many new methods have emerged for clustering single-cell multi-omics data, but typically most of them independently perform dimensionality reduction and then apply the reduced features to the clustering step. This separate two-step approach can lead to suboptimal results.

Here, we propose scMNMF to deal with this problem. scMNMF is an unsupervised method that jointly performs dimensionality reduction and clustering, thereby providing a more accurate and efficient algorithm for cell type discovery. The advantage of this approach is that the feature selection for dimensionality reduction and cell clustering mutually influence each other in an iterative manner, facilitating the discovery of cell types. The results also show that the scMNMF algorithm is more accurate and robust than most algorithms on various datasets.

In the future, the fundamental concepts of scMNMF can be further explored and improved. First, in the data preprocessing step, we can consider operations for addressing dropout phenomena, which can impact clustering performance. Integrating preprocessing, dimensionality reduction and clustering together may lead to improvement. Second, because of the heterogeneity between different omics data, the issue of contribution between different omics data can be considered by assigning them different weights. This will be helpful for our learning process and also one of the directions for further study.

**Key Points**

- The paper provided a novel joint model which introduced dimension reduction, and clustering to the single-cell multi-omics data.
- The paper developed a novel method (scMNMF) and compared the state-of-the-art models of the cell clustering. Aiming at the shortcoming of their models, a novelty model was designed.
- Biological analysis is also conducted to validate the biological significance of our method, including GO, and survival analysis.
- Experimental results have shown that scMNMF has excellent predictive and generalization ability.

## Supplementary Material

scMNMF_support_clean_bbae228

## Data Availability

The data is also accessible from the link.

## Author Biographies

**Yushan Qiu** is an associate professor in Shenzhen University. Her research interest are bioinformatics and machine learning.

**Dong Guo** is a graduate student in Shenzhen University. His research interests include bioinformatics and machine learning.

**Pu Zhao** is an associate professor in Northeastern University. Her majors in cell biology and neurobiology.

**Quan Zou** is a professor in the University of Electronic Science and Technology of China. He is a senior member of IEEE and ACM. He majors in bioinformatics, machine learning and algorithms.
