# Supplementary material for: scMNMF: a novel method for single-cell multi-omics clustering based on matrix factorization
Source: Brief Bioinform. 2024 May 16;25(3):bbae228. doi: 10.1093/bib/bbae228 (PMC11097994; doi:10.1093/bib/bbae228)
Supplement: scMNMF_support_clean_bbae228 [file scmnmf_support_clean_bbae228.pdf]

Support materials

1 Optimization of the scMNMF model

Since the scMNMF objective function is non-convex, we use an iterative strategy to solve it, where one variable is optimized by fixing the other variables, and the iterations continue until the algorithm converges to reach the termination standard. The objective function is as follows

$$\begin{aligned}\mathcal{O} =&R_{W,H_1,\cdots,H_M} + Q_{B,F}\\ =&\sum_{k=1}^{M}\|X_k - WH_k\|^2 + \lambda_k\|H_k\|_1 + \sum_{i=1}^{n}\mu_i\|w_i\|_1\\ &+\|W-BF\|^2 + \alpha\operatorname{Tr}\left(BLB^\top\right)\\ &\text{s.t.} W\geqslant 0, B\geqslant 0, F\geqslant 0.\end{aligned} \tag{1}$$

let Φ_1, Φ_2, Φ_3 be the Lagrange multiplier for constraints W ≥ 0, B ≥ 0, $F \geq 0$, then Lagrange L of scMNMF objective function is

$$L = \mathcal{O} + \operatorname{Tr}\left(\Phi_1 W^T\right) + \operatorname{Tr}\left(\Phi_2 B^T\right) + \operatorname{Tr}\left(\Phi_3 F^T\right).$$

Considering the presence of the L1-norm, here we use the ADMM (Alternating Direction Method of Multipliers) algorithm to optimize this problem. Take into account the Karush-Kuhn-Tucker (KKT) conditions, and then calculating the partial derivatives of W, H_k, B, F, respectively, we have

$$\begin{aligned}\frac{\partial L}{\partial W} =& \left(2\sum_{k=1}^{M}X_kH_k^\top\right) + 2BF + \sigma E + T - \left(2\sum_{k=1}^{M}WH_kH_k^\top\right)\\ &-2W-\sigma W\\ \frac{\partial L}{\partial H_k} =& 2W^\top X_k + \sigma_1 C_k + T_k - 2W^\top WH_k - \sigma_1 H_k\\ \frac{\partial L}{\partial B} =& WF^\top - BFF^\top - \alpha BL\\ \frac{\partial L}{\partial F} =& -2B^\top W + 2B^\top BF\end{aligned} \tag{2}$$

where $\sigma, \sigma_1 > 0$ are penalty parameter, T, T_k are Lagrange multiplier.Then we can get the update rules and summarize the algorithm as follows

Algorithm 1 Optimize our proposed model by alternatingly updating

1: **Input:** single-cell multi-omics data matrix X_k, parameters p, k_1, α.
2: **Initialize:** Initialize W, H_k, B, F, maximum number of iterations and stop error.
3: **Iterate the following processes until convergence:**

$$\begin{aligned}&W \leftarrow W\frac{\left(2\sum_{k=1}^{M}X_kH_k^\top\right) + 2BF + \sigma E + T}{W\left[\left(2\sum_{k=1}^{M}H_kH_k^\top\right) + (2+\sigma)I\right]}\\ &H_k \leftarrow H_k\frac{2W^\top X_k + \sigma_1 C_k + T_k}{2W^\top WH_k + \sigma_1 H_k}\\ &B \leftarrow B\frac{WF^\top}{BFF^\top + \alpha BL}\\ &F \leftarrow F\frac{B^\top W}{B^\top BF}\\ &E = S_{\mu/\sigma}\left(W - \frac{T}{\sigma}\right)\\ &T = T + \sigma(E - W)\\ &C_k = S_{\lambda_k/\sigma_1}\left(H_k - \frac{T_k}{\sigma_1}\right)\\ &T_k = T_k + \sigma_1\left(C_k - H_k\right)\end{aligned}$$

where $\operatorname{Soft}(x) := \operatorname{sgn}(x)(|x| - y)_+, (x)_+ = \max(x, 0)$
4: **Output:** W, H_k, B, F

2 Convergence analysis

We conducted a convergence analysis on the iterative formulas of each variable obtained by the alternating iterative method. For each variable, we discussed its convergence and attempted to find the optimal solution. Here, we will only discuss W, and the same applies to $H_k, B, andF$. Construct a auxiliary function $G(W, W^t)$, let diagonal matrix

$$L_{ij}\left(W^t\right) = \delta_{ij}\frac{\left[2\sum_{k=1}^{M}WH_kH_k^\top + (2+\sigma)W\right]_{pi}}{W_{pi}^t} \tag{3}$$

then

$$\begin{aligned}G\left(W, W^t\right) =& F\left(W^t\right) + \left(W - W^t\right)^T\nabla_W F\left(W^t\right)\\ &+\frac{1}{2}\left(W - W^t\right)L\left(W^t\right)\left(W - W^t\right).\end{aligned}$$

take the second-order Taylor expansion of $F(W)$.

$$\begin{aligned}F(W) =& F\left(W^t\right) + \left(W - W^t\right)^\top\nabla_W F\left(W^\top\right)\\ &+\frac{1}{2}\left(W - W^t\right)^\top\nabla_W^2 F\left(W^t\right)\left(W - W^t\right).\end{aligned}$$

now we only need prove $G\left(W, W^t\right) \geqslant F(W)$, then $G\left(W, W^t\right)$ is the auxiliary function of $F(W)$.

$$\nabla_W^2 F(W) = \frac{\partial\nabla_W F(W)}{\partial W^\top} = 2\sum_{k=1}^{M}H_kH_k^\top + (2+\sigma)I.$$

then

$$\begin{aligned}&G\left(W, W^t\right) - F(W)\\ =&\frac{1}{2}\left(W - W^t\right)^\top\left[L\left(W^t\right)\left(W - W^t\right) - \nabla_W^2 F\left(W^t\right)\left(W - W^t\right)\right].\end{aligned}$$

let

$$M_{ij}\left(W^t\right) = W_{ij}\left[L\left(W^t\right) - \nabla_W^2 F\left(W^t\right)\right]_{ij}W_j^t.$$

The following is a proof that matrix M_{ij} is positive semi-definite.

$$\begin{aligned}\nu^{\top} M\nu &= \sum_{ij} v_i M_{ij} v_j \\ &= \sum_{ij} v_i W_i^t L\left(W^t\right)_{ij} W_j^t v_j - v_i W_i^t \nabla_W^2 F\left(W^t\right)_{ij} W_j^t v_j \\ &= \sum_{ij} 2\sum_{k=1}^{M}\left(H_k H_k^{\top}\right)_{ij} W_i^t W_j^t v_i^2 + \sum_{ij}(2+\sigma) W_i^t W_j^t v_i^2 \\ &- \sum_{ij} 2\sum_{k=1}^{M}\left(H_k H_k^{\top}\right)_{ij} W_i^t W_j^t v_i v_j - \sum_{ij}(2+\sigma) W_i^t W_j^t v_i^2 \\ &= \sum_{k=1}^{M}\sum_{ij}\left(H_k H_k^{\top}\right)_{ij} W_i^t W_j^t \left(v_i - v_j\right)^2 \geqslant 0.\end{aligned}$$

for

$$\frac{\partial G\left(W \cdot W^t\right)}{\partial W} = 0$$

we have

$$\begin{aligned}W^{t+1} &= W^t - L^{-1}\left(W^t\right)\nabla_W\left(F\left(W^t\right)\right. \\ &\leftarrow W^t \frac{2\sum_{k=1}^{M} X_k H_k^{\top} + 2BF + \sigma E + T}{2\sum_{k=1}^{M} W H_k H_k^{\top} + (2+\sigma)W}.\end{aligned}$$

Consequently, it converges in accordance with the iterative process.

3 Parameter selection

The parameters p, k_1 represent the number of latent variables used for dimension reduction and the number of clusters (i.e., the number of cell types). For the parameter p, we use the instability-based NMF model to calculate the specific value. scMNMF algorithm runs t times with random initial solutions and gets t basis matrices, denoted as $M_1, \cdots, M_t$. Support two matrices M_1 and M_2, a $t \times t$ matrix G is defined where the element g_{ij} is the cross correlation between the $i\text{-}th$ column of the matrix M_1 and the $j\text{-}th$ column of matrix M_2. Then the dissimilarity between M_1 and M_2 is defined as

$$\operatorname{diss}\left(M_1, M_2\right) = \frac{1}{2p}\left(2p - \sum_j \max g_{\cdot j} - \sum_i \max g_{i \cdot}\right)$$

Where $g_{\cdot j}$ denotes the $j\text{-}th$ column of matrix G. The instability is the discrepancy of all the basis matrices for p, which is defined as

$$\gamma(p) = \frac{2}{t(t-1)}\sum_{1 \leq i \leq j \leq t} \operatorname{diss}\left(M_1, M_2\right)$$

The p corresponding to the minimal $\gamma(p)$ is selected as the number of rows in G. For the parameters k_1, considering partial label information is known, then the number of clusters can be directly taken as the value of parameter k_1. We use cross validation to find the optimal values for μ_i, however, all μ_i have been set to 0.5 in the experiment to save computational time since the the clustering performance is insensitive to the parameter. The parameters λ_k and α are used to balance the strength of the fitting term and the regularization term. Similar to the DRjCC model, we define

the values of the two parameters as follows:

$$\lambda_k = \frac{\|X_k\|_2}{\|H_k\|_2}, \quad \alpha = \frac{\|W\|_2}{\|B\|_2}. \tag{4}$$

Then λ_k and α are automatically updated according to this formula.

4 Data pre-processing

Before the experiment, we preprocessed the input single-cell multi-omics data matrix X_k with the following steps:

1. Delete genes that are not expressed on all cells..
2. Filtering out genes that are only present in a small number of cells.
3. Log transformation and normalization.

5 Informative gene selection

The selection of informative genes involves determining how many informative genes to choose and how to extract them. Here are the steps we take to select informative genes:

1. Normalize the matrix H such that the mean of each row is 0.
2. Calculate the eigenvalues and corresponding eigenvectors of the matrix HH^T. Arrange them in descending order of eigenvalues, denoted as $\lambda_1 > \cdots > \lambda_n$ and $\mathbf{y}_1, \ldots, \mathbf{y}_n$ respectively.
3. Select the number k of informative genes: For the selection of k, we choose the value of k such that the cumulative contribution of the largest k eigenvalues is greater than a threshold δ, i.e., $k = \arg_l \sum_{i=1}^{l} \lambda_i / \sum_{i=1}^{n} \lambda_i \geq \delta$. Here, we set the threshold δ to 0.95.
4. Define the weight of the i-th gene as $\beta_i = \sum_{j=1}^{k} \lambda_j y_{ji}$, and select the top k genes based on the weights in descending order.

We selected 28 informative genes from the 10X_10K dataset using this approach, which refer to Table 1 for the specific gene names.

6 Support table and figures

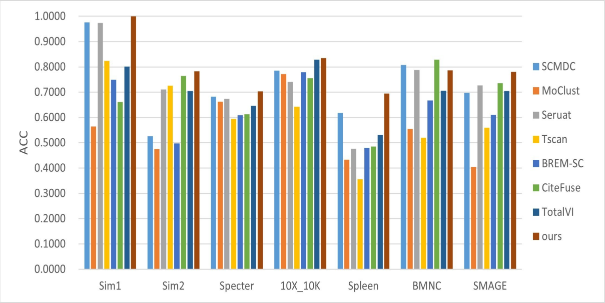

(a)

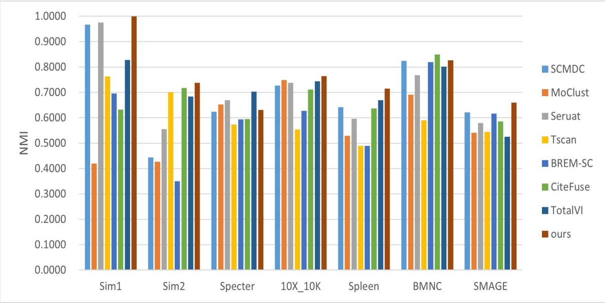

(b)

Fig. 1. ACC, NMI of different algorithms on 2 simulated datasets and 5 real single-cell multi-omics datasets.

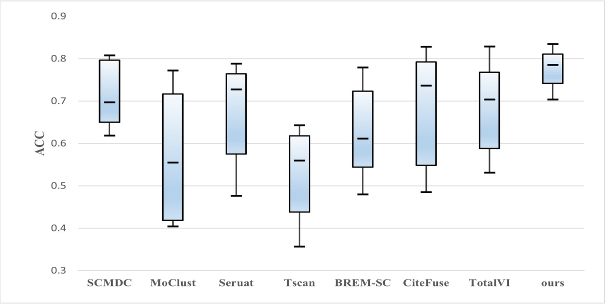

(a)

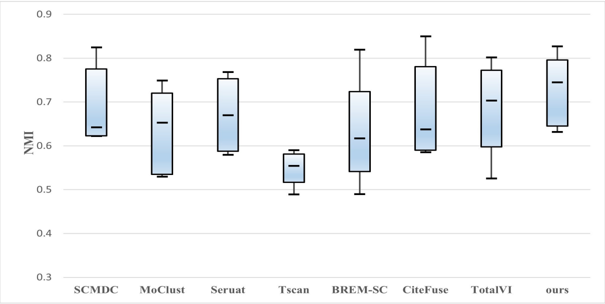

(b)

Fig. 2. Boxplots of different algorithms on 5 real single-cell multi-omics datasets.

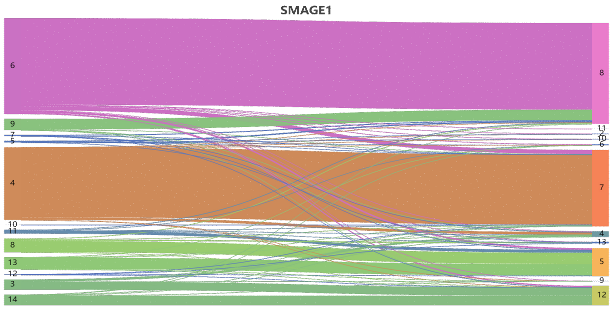

(a)

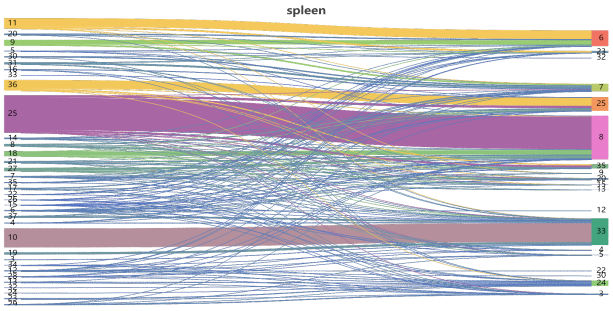

(b)

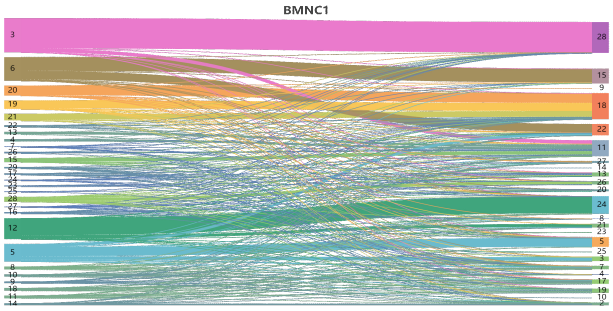

(c)

Fig. 3. Sankey plots: (a)"SMAGE dataset", (b)"Spleen dataset", (c)"BMNC dataset".

Table 1. Maker gene for 10X_10K dataset, where the bolded genes indicated a significant correlation with the survival time of patients.

ACOT7	YBX1	DEPDC1	MYL9
CDC20	GATA2	FCGR2B	ORC1
FANCI	RRM2	CDCA8	PRDX1
C1QB	HELLS	NASP	FCGR2B
OSTC	DDOST	CKS1B	RPN2
SPARC	GP9	LAMP5	WARS
UGCG	IGHV3-30	PAICS	KIF23

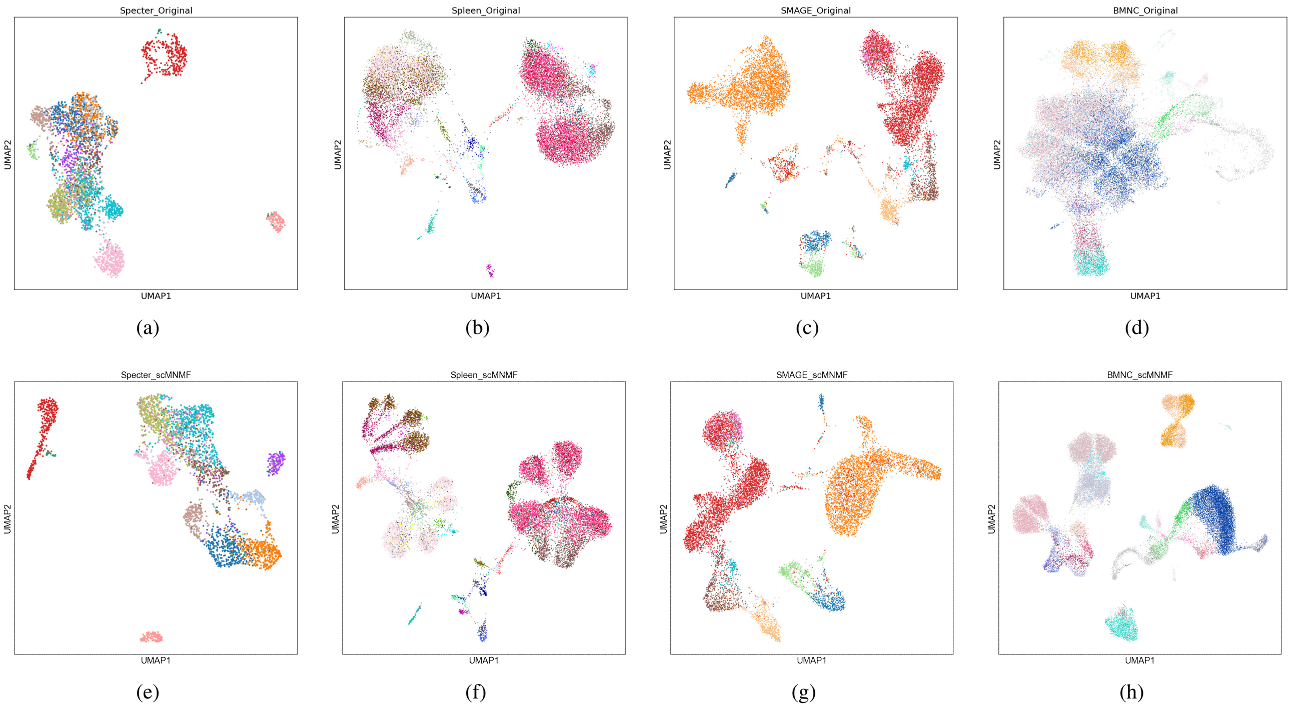

Fig. 4. Visualization of cell clusters before and after dimension reduction of the other four datasets.

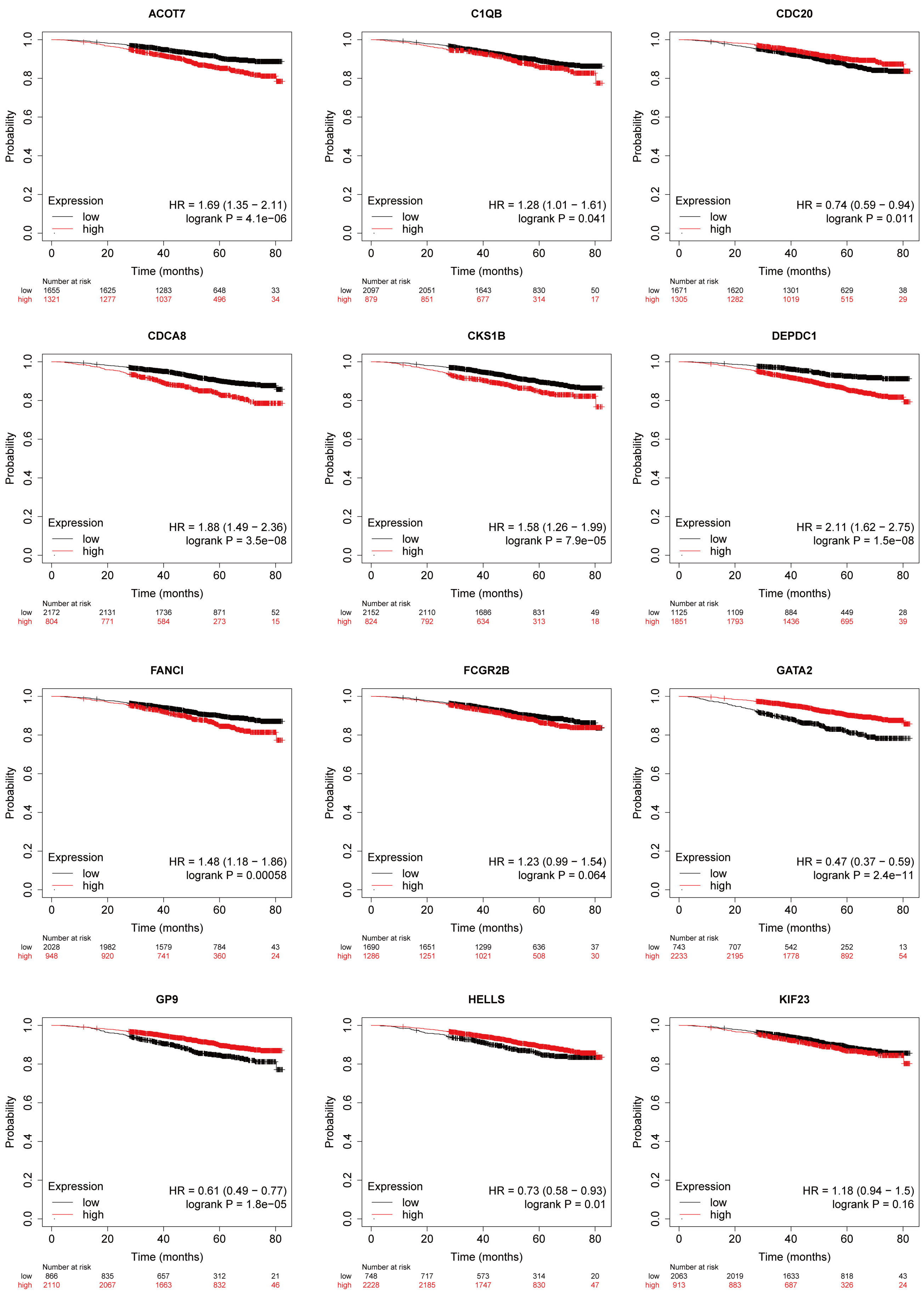
ACOT7
HR = 1.69 (1.35 − 2.11)
logrank P = 4.1e−06
Number at risk
low 1655 1625 1283 648 33
high 1321 1277 1037 496 34
C1QB
HR = 1.28 (1.01 − 1.61)
logrank P = 0.041
Number at risk
low 2097 2051 1643 830 50
high 879 851 677 314 17
CDC20
HR = 0.74 (0.59 − 0.94)
logrank P = 0.011
Number at risk
low 1671 1620 1301 629 38
high 1305 1282 1019 515 29
CDCA8
HR = 1.88 (1.49 − 2.36)
logrank P = 3.5e−08
Number at risk
low 2172 2131 1736 871 52
high 804 771 584 273 15
CKS1B
HR = 1.58 (1.26 − 1.99)
logrank P = 7.9e−05
Number at risk
low 2152 2110 1686 831 49
high 824 792 634 313 18
DEPDC1
HR = 2.11 (1.62 − 2.75)
logrank P = 1.5e−08
Number at risk
low 1125 1109 884 449 28
high 1851 1793 1436 695 39
FANCI
HR = 1.48 (1.18 − 1.86)
logrank P = 0.00058
Number at risk
low 2028 1982 1579 784 43
high 948 920 741 360 24
FCGR2B
HR = 1.23 (0.99 − 1.54)
logrank P = 0.064
Number at risk
low 1690 1651 1299 636 37
high 1286 1251 1021 508 30
GATA2
HR = 0.47 (0.37 − 0.59)
logrank P = 2.4e−11
Number at risk
low 743 707 542 252 13
high 2233 2195 1778 892 54
GP9
HR = 0.61 (0.49 − 0.77)
logrank P = 1.8e−05
Number at risk
low 866 835 657 312 21
high 2110 2067 1663 832 46
HELLS
HR = 0.73 (0.58 − 0.93)
logrank P = 0.01
Number at risk
low 748 717 573 314 20
high 2228 2185 1747 830 47
KIF23
HR = 1.18 (0.94 − 1.5)
logrank P = 0.16
Number at risk
low 2063 2019 1633 818 43
high 913 883 687 326 24
Expression
low
high
Probability
Time (months)

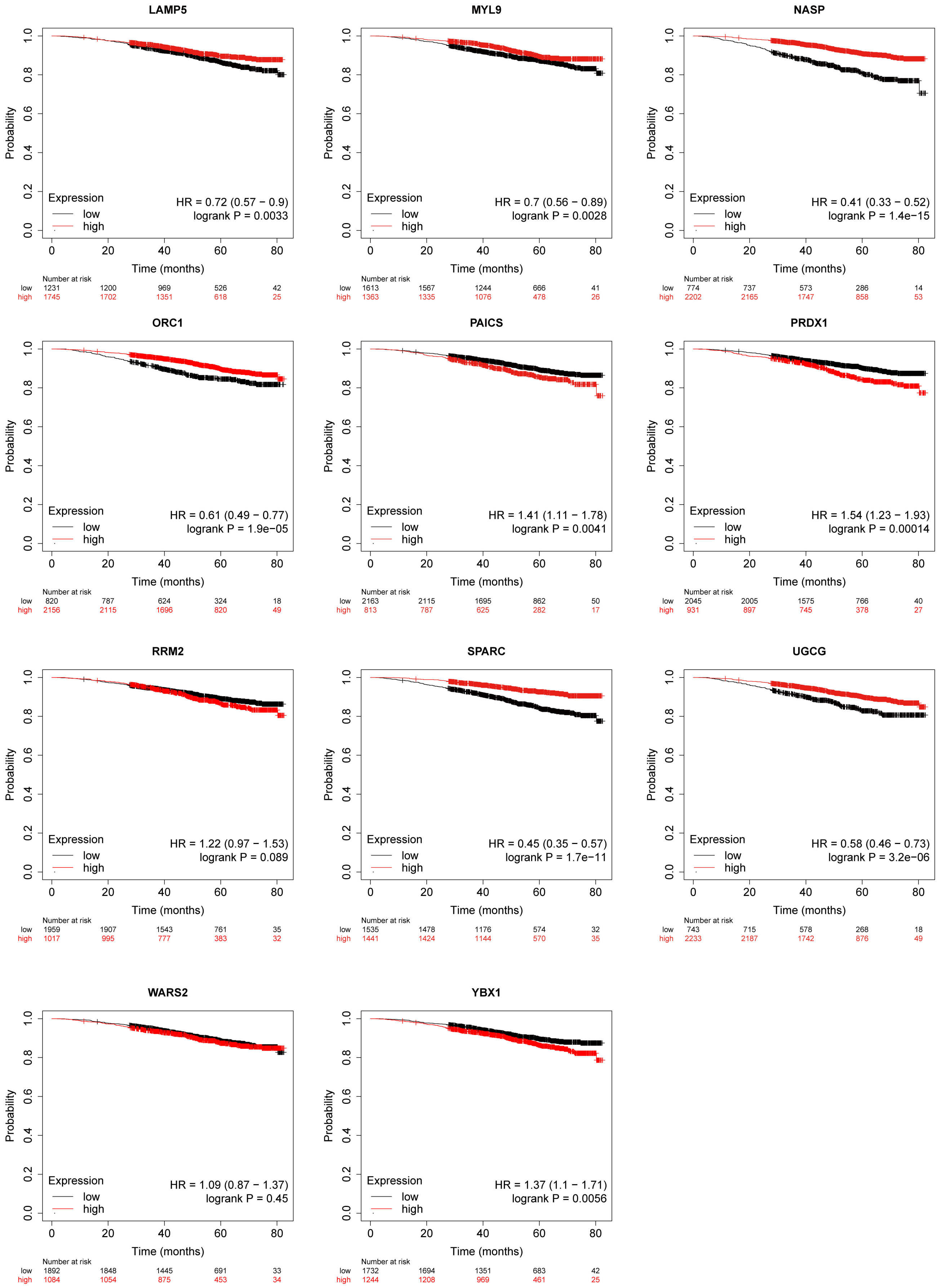

Fig. 5. Kaplan–Meier survival analysis of marker genes.
